# Effects of a Fish Oil Rich in Docosahexaenoic Acid on Cardiometabolic Risk Factors and Oxidative Stress in Healthy Rats

**DOI:** 10.3390/md19100555

**Published:** 2021-09-29

**Authors:** Bernat Miralles-Pérez, Lucía Méndez, Maria Rosa Nogués, Vanessa Sánchez-Martos, Àngels Fortuño-Mar, Sara Ramos-Romero, Mercè Hereu, Isabel Medina, Marta Romeu

**Affiliations:** 1 Functional Nutrition, Oxidation and Cardiovascular Diseases Research Group (NFOC-SALUT), Pharmacology Unit, Department of Basic Medical Sciences, Universitat Rovira i Virgili, C/Sant Llorenç 21, E-43201 Reus, Spain; bernat.miralles@urv.cat (B.M.-P.); vanessa.sanchez@urv.cat (V.S.-M.); marta.romeu@urv.cat (M.R.); 2 Chemistry of Marine Products, Department of Food Technology, Institute of Marine Research (IIM-CSIC), C/Eduardo Cabello 6, E-36208 Vigo, Spain; luciamendez@iim.csic.es (L.M.); medina@iim.csic.es (I.M.); 3 Eldine Patología, C/Plom 32, E-43006 Tarragona, Spain; afortunyo@eldinepatologia.org; 4 Department of Biological Chemistry, Institute of Advanced Chemistry of Catalonia (IQAC-CSIC), C/Jordi Girona 18-26, E-08034 Barcelona, Spain; sara.ramosromero@ub.edu (S.R.-R.); merce.hereu@iqac.csic.es (M.H.); 5 Department of Cell Biology, Physiology & Immunology, Faculty of Biology, University of Barcelona, Avd/Diagonal 643, E-08028 Barcelona, Spain

**Keywords:** eicosapentaenoic acid, soybean oil, coconut oil, cholesterol, glucose, inflammation, lipid peroxidation, protein carbonylation

## Abstract

Omega-3 polyunsaturated fatty acids are associated with a lower risk of cardiometabolic diseases. However, docosahexaenoic acid (DHA) is easily oxidized, leading to cellular damage. The present study examined the effects of an increased concentration of DHA in fish oil (80% of total fatty acids) on cardiometabolic risk factors and oxidative stress compared to coconut oil, soybean oil, and fish oil containing eicosapentaenoic acid (EPA) and DHA in a balanced ratio. Forty healthy male Sprague–Dawley rats were supplemented with corresponding oil for 10 weeks. Supplementation with the fish oil containing 80% DHA decreased plasma fat, plasma total cholesterol and muscle fat compared to the coconut oil and the soybean oil. Increasing concentrations of DHA induced incorporation of DHA and EPA in cell membranes and tissues along with a decrease in ω-6 arachidonic acid. The increase in DHA promoted lipid peroxidation, protein carbonylation and antioxidant response. Taken together, the increased concentration of DHA in fish oil reduced fat accumulation compared to the coconut oil and the soybean oil. This benefit was accompanied by high lipid peroxidation and subsequent protein carbonylation in plasma and in liver. In our healthy framework, the slightly higher carbonylation found after receiving fish oil containing 80% DHA might be a protecting mechanism, which fit with the general improvement of antioxidant defense observed in those rats.

## 1. Introduction

Cardiovascular diseases (CVD), such as ischemic heart disease and stroke, are major causes of death worldwide [1]. Risk factors for CVD include dyslipidemia, obesity, diabetes mellitus and increased blood pressure [2]. These CVD risk factors are highly related to dietary pattern. Observational data have shown that high intake of saturated fatty acids (SFA) increases the risk for CVD [3], whereas unsaturated fatty acids decrease that risk [4]. Therefore, the replacement of SFA by unsaturated fatty acids might be a useful nutritional strategy against metabolic alterations and CVD events [5,6].

Supplementation with omega-3 polyunsaturated fatty acids (ω-3 PUFA), specifically eicosapentaenoic acid (EPA, 20:5) and docosahexaenoic acid (DHA, 22:6), exhibits cardioprotective effects by modulating lipid metabolism, vascular function, cell membrane dynamics as well as anti-inflammatory and antioxidant responses [7,8]. The use of fish oil supplements is prevalent in Western societies [9]. Importantly, the supplements can differ in composition of ω-3 PUFA, providing EPA and DHA individually or in combination. Circulating and tissue lipids reflect the dietary fat intake [10,11]. Thus, diets enriched with fish oil increase the amount of ω-3 PUFA and related oxidized metabolites such as eicosanoids and docosanoids in rodents compared to soybean oil rich in linoleic acid (ω-6 LA; 18:2) [10,11]. Low ω-6/ω-3 ratio has been associated with a decrease in inflammation [12] and atherosclerotic lesions [13]. Furthermore, previous studies [10,11,14,15,16,17,18] have shown in rats that supplementation with fish oil decreases blood glucose, glycated hemoglobin, plasma total fatty acids, pro-inflammatory mediators and oxidative stress compared to soybean oil. In rodents, fish oil containing EPA/DHA in a balanced ratio (1:1) induces better health outcomes in blood glucose, pro-inflammatory mediators in tissues and oxidative damage to proteins than unbalanced EPA/DHA ratios of 2:1 and 1:2 [16,17,18]. When provided individually in humans, DHA may promote greater decrease in pro-inflammatory mediators and circulating lipids than EPA [19].

Various mechanisms are involved in promoting the beneficial effects on cardiometabolic health resulting from fish oil supplements. First, ω-3 PUFA exert hypolipidemic effect through the activation of the peroxisome proliferator-activated receptor (PPAR)α, which in turn increases fatty acid β-oxidation and cholesterol uptake from plasma as well as inhibiting lipogenesis [15,20,21]. Second, ω-3 PUFA exert an anti-inflammatory effect by binding to G-protein-coupled receptor 120 and increasing PPARγ, inhibiting the nuclear factor-kappa β (NF-κβ) pro-inflammatory signaling pathway [22,23]. The ω-3 and ω-6 PUFA—e.g., arachidonic acid (ARA)—may also compete for enzymes such as phospholipases A2, cyclooxygenases (COX) and lipoxygenases (LOX) [24]. In this manner, oxygenated lipids derived from ω-3 PUFA by COX and LOX action, such as resolvins, protectins, and maresins, exhibit anti-inflammatory and pro-resolving properties [24]. Furthermore, eicosanoids derived from EPA induce lower inflammatory responses than those from ω-6 PUFA [25]. Finally, ω-3 PUFA exert antioxidant effect through the induction of the nuclear factor-erythroid 2-related factor 2 (Nrf2) signaling pathway, increasing the endogenous antioxidant response [26].

However, because PUFA are highly susceptible to oxidation [27], supplementation with ω-3 PUFA may exhibit beneficial or deleterious effects in a dose-dependent manner [28,29]. Lipid peroxidation end products, such as 4-hydroxyhexenal (HHE), 4-hydroxynonenal (4-HNE) and malondialdehyde (MDA) derived from ω-3 and ω-6 PUFA, might form adducts with proteins leading to alterations in cell signaling and metabolic pathways [30]. In fact, accumulation of oxidized lipids is associated with increased liver triacylglycerol [29] inflammation [28] and CVD [31]. Lipid peroxidation is highly dependent on the degree of unsaturation in a fatty acid [27]. Thus, DHA might be more vulnerable to peroxidation than other fatty acids.

We hypothesized that increased concentration of DHA in fish oil beneficially affects cardiometabolic risk factors and oxidative stress in healthy individuals. The aim of this study was to examine the effects of an increased concentration of DHA in fish oil compared to coconut oil as source of saturated fat, soybean oil as source of unsaturated fat particularly ω-6 LA, and fish oil containing EPA/DHA (1:1) on cardiometabolic risk factors and oxidative stress in healthy male rats.

## 2. Results and Discussion

### 2.1. Biometric Data, Glucose Metabolism, Plasma Lipid Profile, Ectopic Fat and Transaminases

**Figure 1:**
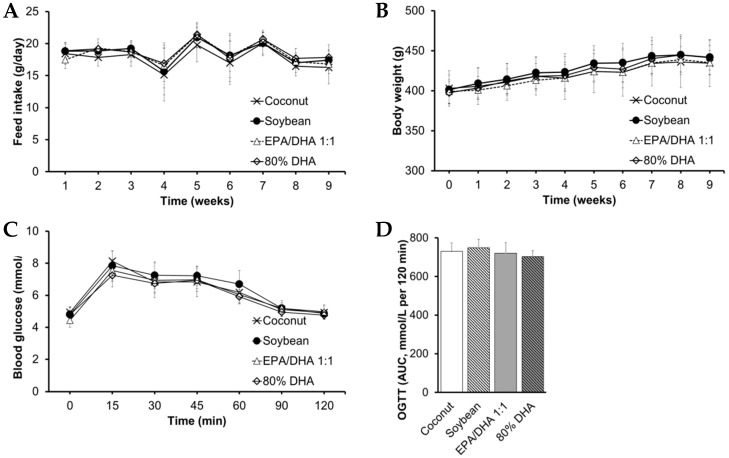
Feed intake (**A**), biometric data (**B**) and oral glucose tolerance at week 9 (**C**,**D**). Abbreviations: EPA, eicosapentaenoic acid; DHA, docosahexaenoic acid; OGTT, oral glucose tolerance test; AUC, area under the curve. OGTT was performed during week nine and the area under the curve (mmol/L per 120 min) was calculated using the Trapezium method. No significant differences were found (*p*-value > 0.05).

Fish oil containing 80% DHA as well as fish oil containing EPA/DHA 1:1 and coconut oil decreased fasting blood glucose in contrast to soybean oil at the end of the study (Table 1 and Table S1). Nevertheless, we observed no significant differences in either glucose tolerance—i.e., oral glucose tolerance tests (OGTT), feed intake or biometric data among groups (Figure 1 and Table S1). These findings agree with our previous study in healthy female Wistar rats [17]. In the present study, we administered a higher dose of fish oil than that used in our previous studies in healthy rats [16,17,18] with the aim of achieving a high incorporation of DHA into cell membranes and tissues. Other authors [21,32] have shown that supplementation with ω-3 PUFA decreases circulating glucose and enhances insulin sensitivity in tissues via action of adiponectin and PPARs. Interestingly, we observed no differences in parameters of glucose metabolism between rats supplemented with the fish oil containing either 80% DHA or EPA/DHA 1:1 and those supplemented with the coconut oil (Figure 1 and Table 1). A previous study [33] has shown that adaptive mechanisms such as increased fatty acid β-oxidation as well as decreased lipogenesis and gluconeogenesis might explain why glucose homeostasis is maintained after either high intake of SFA or PUFA under isocaloric conditions. Nevertheless, soybean oil increased blood glucose compared to the coconut oil (Table 1). Other authors [34] have suggested that supplementation with virgin coconut oil may beneficially modulate blood glucose probably via action of medium-chain FA lauric acid and polyphenols. Furthermore, other authors [35] have shown that a supplementation with soybean oil inhibits the PI3K/AKT signaling pathway, which may contribute to explain the differences in glucose homeostasis among groups.

The supplementation with fish oil containing either 80% DHA or EPA/DHA 1:1 clearly modulated total fat content in plasma (Table 1). Fish oil containing 80% DHA also decreased plasma total cholesterol (TC) mainly from high-density lipoprotein cholesterol (HDL) compared to both the coconut oil and the soybean oil (Table 1). Furthermore, fish oil containing EPA/DHA 1:1 decreased plasma HDL compared to the coconut oil. In contrast, the rats supplemented with soybean oil had the most beneficial low-density lipoprotein cholesterol (LDL)/HDL ratio (Table 1). Hypercholesterolemia is a well-known risk factor for CVD [2]. Previous studies [36,37] have shown that ω-3 PUFA might decrease plasma cholesterol by stimulating of reverse cholesterol transport via an increase in scavenger receptor class B-1 expression and subsequent excretion of cholesterol by the liver. In the present study, fish oil containing 80% DHA even significantly decreased plasma HDL compared to EPA/DHA 1:1 (Table 1). In agreement with our results, it has been suggested that DHA is a more potent agent for lowering plasma lipids than EPA [7,19]. Other authors [38] have shown that a fish oil with EPA/DHA 1:2 might exert higher hypocholesterolemic effect than a balanced ratio in male apolipoprotein E knockout C57/BL6 mice on high-fat diet. Our previous studies [10,17,18] have shown no differences among distinct EPA/DHA ratios (2:1, 1:1 or 1:2) in healthy female Wistar rats. In the present study, the increase in LDL/HDL ratio after receiving the fish oil containing either 80% DHA or EPA/DHA 1:1 mainly compared to the soybean oil (Table 1) could be also explained by an increase in LDL particle size as suggested by other authors [39]. It has been suggested that large LDL particles exhibit less harmful effects compared to small LDL particles [40]. Nevertheless, a recent study has evidenced an opposite effect of ω-3 PUFA supplements on LDL size [41]. Further investigation is required to understand the influence of ω-3 PUFA on lipoprotein properties. Although several studies [19,41] have shown that fish oil decreases plasma triacylglycerol (TAG) concentration, we observed no differences in plasma TAG among groups (Table 1). Our results are consistent with the observations of other studies in animal models [17,29].

In the present study, the supplementation with fish oil containing either 80% DHA or EPA/DHA 1:1 influenced ectopic fat accumulation (Table 1). Thus, fish oil containing 80% DHA decreased total fat content in gastrocnemius muscle compared to both coconut oil and soybean oil. On the other hand, fish oil containing 80% DHA as well as the coconut oil increased total fat content in the liver compared to the fish oil containing EPA/DHA 1:1. Nevertheless, no significant differences in liver TAG, TC and histological data were found among groups (Table 1, Figure S1A). A previous study [29] have shown that a high dose of DHA induces accumulation of TAG in the liver of hypercholesterolaemic New Zealand White male rabbits, probably via an increase in lipoprotein receptors VLDLR and LDLR regulated by Farnesol X receptor. Interestingly, fish oil containing 80% DHA tended to increase plasma aspartate aminotransferase (AST) activity mainly compared to the soybean oil without affecting the ratio of AST to alanine aminotransferase ALT (Table 1). No differences in plasma AST/ALT ratio and in liver histology (Table 1, Figure S1A) indicated no impaired liver function. AST can be released into circulation by other tissues, such as muscle. The tendency for an increase in plasma AST may be related with the lower muscle fat content observed in those animals supplemented with high doses of DHA. However, further investigation is required to understand the effect of increased concentration of DHA in fish oil on transaminases and muscle function.

### 2.2. Fatty Acid Composition of Erythrocytes and Tissues

Erythrocytes are considered more robust markers of ω-3 PUFA intake than plasma [42]. In the present study, the supplementation with the fish oils containing either 80% DHA or EPA/DHA 1:1 increased the total amount of ω-3 PUFA and decreased the total amount of ω-6 PUFA in erythrocytes, perigonadal adipose tissue and liver compared to both coconut oil and soybean oil. In particular, fish oil containing 80% DHA induced the lowest ratio of ω-6 to ω-3 compared to all the other oils (Table 2, Table 3 and Table 4).

In accordance with the composition of the oils (Table S2), the supplementation with fish oil containing 80% DHA promoted higher incorporation of DHA in erythrocytes, perigonadal adipose tissue and liver than all the other oils (Table 2, Table 3 and Table 4). Fish oil containing 80% DHA provided lower amount of EPA than fish oil containing EPA/DHA 1:1 (Table S2). The group supplemented with the fish oil containing 80% DHA consumed average doses of DHA and EPA of 173 mg and 8 mg per kg body weight per day, respectively, whereas the group supplemented with the fish oil containing EPA/DHA 1:1 consumed average doses of DHA and EPA of 55 mg and 58 mg, respectively. In spite of this, the two types of fish oil induced similar incorporation of EPA in erythrocytes and in liver (Table 2 and Table 4). Our results are consistent with the observations of other authors [43]. Considering the pathways of EPA formation, fish oil containing 80% DHA seemed to promote elongation and desaturation (Δ5/6D) from α-linolenic acid (ALA, 18:3 ω-3) to EPA. Furthermore, the conversion from EPA to docosapentaenoic acid (DPA, 22:5 ω-3) may be reduced by the supplementation of the fish oil containing 80% DHA. Thus, the modulation of these two steps after receiving the fish oil containing 80% DHA resulted in high incorporation of EPA in erythrocytes and liver. In agreement with these results, a previous study [44] showed that high dietary intake of ω-3 PUFA in the Inuit population decreased the conversion pathway from EPA to DPA, without significant effect on DHA. This fact has been considered a mechanistic adaptation to compensate for a high dietary intake of EPA [44]. This effect of the supplementation with the fish oil containing 80% DHA on EPA were not found in perigonadal adipose tissue (Table 3).

The supplementation with fish oils containing either 80% DHA or EPA/DHA 1:1 decreased the amount of ω-6 PUFA—i.e., ARA (20:4), docosatetraenoic acid (DTA, 22:4) and docosapentaenoic acid (DPA, 22:5)—in erythrocytes and in liver compared to the coconut oil and the soybean oil (Table 2 and Table 4) but not in the perigonadal adipose tissue (Table 3). Importantly, the two types of fish oil provided higher amounts of ARA, DTA and DPA than either coconut oil or soybean oil (Table S2). The decrease in these ω-6 PUFA in erythrocytes and in liver might be expected, given that the precursors of ω-3 and ω-6 PUFA pathways compete for the same desaturase enzymes [45]. In consonance with this, the supplementation with fish oil containing either 80% DHA or EPA/DHA 1:1 decreased the activity of Δ5-desaturase in the ω-6 PUFA pathway, resulting in a low conversion from dihomo-γ-linolenic acid (DGLA, 20:3 ω-6) to ARA. Furthermore, the activity of Δ6-desaturase = [20: 3 ω-6/18: 2 ω-6] remained unchanged (Table 2, Table 3 and Table 4). Taken together, these results showed that the supplementation with the fish oil containing either 80% DHA or EPA/DHA 1:1 modulate the ω-6 PUFA pathway, resulting in an accumulation of precursors—both LA and DGLA—along with a decrease in ARA, DTA and DPA (Table 2 and Table 4).

As regards to stearoyl-CoA desaturase-1 (SCD-1) indexes, fish oil containing 80% DHA decreased values of SCD-16 in erythrocytes and in liver, but not those of SCD-18, compared to the coconut oil (Table 2 and Table 4). SCD-16 and SCD-18 catalyze desaturation from palmitic acid (16:0) to palmitoleic acid (16:1 ω-7) and stearic acid (18:0) to oleic acid (18:1 ω-9), respectively. Low activities of SCD-1 may be associated with low accumulation of fat, low inflammation and prevention of obesity [46].

Finally, fish oil containing 80% DHA as well as fish oil containing EPA/DHA 1:1 and soybean oil induced lower incorporation of myristic acid (14:0) in perigonadal adipose tissue than coconut oil (Table 3). Despite the distinct fatty acid composition between coconut oil and soybean oil (Table S2), there were no other major differences in the fatty acid composition of their corresponding erythrocytes, perigonadal adipose tissue and liver (Table 2, Table 3 and Table 4).

### 2.3. Membrane Fluidity of Erythrocytes

Although highly distinct fatty acid composition of erythrocytes among groups (Table 2), no significant differences in membrane fluidity were found (Table 1). It has been suggested that the fatty acid composition and the cholesterol content of membrane modulates fluidity, ion permeability and protein function [47]. It is assumed that increasing polyunsaturated fatty acids in membrane lead to an increase in its fluidity [47]. In the present study, no differences in total content of PUFA in erythrocytes may explain why the membrane fluidity remained unchanged.

### 2.4. Biomarkers of Inflammation and Oxidative Stress

The supplementation with fish oil containing either 80% DHA or EPA/DHA 1:1 promoted a small increase in plasma tumor necrosis factor α (TNFα) concentration compared to both coconut oil and soybean oil (Table 1). Despite that, we observed no significant differences in either plasma C-reactive protein (CRP) concentration (Table 1) or presence of portal chronic inflammation in the liver among groups (Figure S1B). TNFα is highly associated with CRP [48]. Nevertheless, it is important to note that concentration of plasma TNFα remained at normal, very low values in all the rats of the present study. Thus, secretion of CRP into plasma was not modified in our healthy framework. Importantly, both low ω-6/ω-3 ratio and low SCD-16 activity are associated with a beneficial inflammatory status [12,46]. In agreement with our results, it has been suggested that ω-3 PUFA supplements decrease TNFα production inhibitor prostaglandin E2 [8], which is derived from arachidonic acid. The increase in plasma TNFα may be due to a low TNFα expression in rats supplemented with the coconut oil and the soybean oil rather than an increase in those supplemented with two type of fish oil. In contrast, other studies have shown that ω-3 PUFA might increase secretion of TNFα from resident macrophages in adipose tissue [49] and TNFα gene expression in adipocytes [28,50].

As far as oxidative stress is concerned, the fish oil containing 80% DHA markedly increased the amount of thiobarbituric acid-reactive substances (TBARS) in plasma, erythrocytes and perigonadal adipose tissue (Table 5 and Table 6). In fact, lipid peroxidation was dependent on the degree of unsaturation of the oils—80% DHA > EPA/DHA 1:1 > soybean > coconut. Furthermore, fish oil containing 80% DHA increased the abundance of protein carbonyls in plasma compared to both the coconut oil and the soybean oil (Table 5), and in the liver compared to the soybean oil (Table 6). Nevertheless, no significant differences were observed in either the oxidized LDL in plasma (Table 5) or the conjugated diene hydroperoxides in perigonadal adipose tissue and in the liver (Table 6). The TBARS assay estimates the amount of end products of lipid peroxidation, which can react with proteins [30]. The most abundant protein in plasma is albumin [51]. Albumin exhibits antioxidant properties by enzymatic activity and by scavenging of reactive compounds [52]. In the liver, a previous study [14] have shown that actin is one of the main carbonylated proteins after receiving fish oil supplements. Like albumin, actin may act as scavenger without undergoing significant functional impairment [53]. Thus, lipid peroxidation ensuing from the dietary intake of fish oil containing 80% DHA leads to protein carbonylation of plasma and liver proteins. Possibly, the carbonylation of abundant proteins, such as albumin and actin, in plasma and liver, respectively [14], may prevent the oxidative damage to other critical proteins, such as LDL particles in plasma, under high exposure to unsaturated fat.

Concurrently, the group supplemented with fish oil containing 80% DHA had the highest value of oxygen radical absorbance capacity (ORAC) among groups (Table 5). Importantly, the high value of ORAC may be associated with a high antioxidant activity of albumin in blood samples [54]. This observation was accompanied by an increase in erythrocyte glutathione peroxidase (GPx) activity compared to both soybean oil and fish oil containing EPA/DHA 1:1 (Table 5), and a decrease in adipocyte glutathione reductase (GR) activity compared to the soybean oil (Table 6). Furthermore, liver oxidized glutathione (GSSG) and liver xanthine oxidase (XO) tended to decrease after receiving fish oil containing 80% DHA compared to coconut oil and soybean oil, respectively (Table 6). Other authors [55] have shown that high activity of XO in serum is associated with high risk for developing diabetes. We observed no significant differences in either cytosolic superoxide dismutase (SOD), catalase (CAT), GPx, glutathione S-transferase (GST) or reduced glutathione (GSH) in perigonadal adipose tissue and in liver among groups. However, we cannot exclude differences in other critical enzymes related to antioxidant defense (e.g., heme oxygenase-1), in other cell compartments and in other tissues. In fact, high content of DHA in cell membranes may promote an increased antioxidant response through the action of lipid peroxidation end products [56]. In agreement with our observations, previous studies [29,43] have shown that supplementation with ω-3 PUFA increases the amount of TBARS, 4-HHE and 4-HHE-protein adducts in a dose-dependent manner. Furthermore, Takahashi et al. [57] showed that a very high dose of fish oil induces gene expression of enzymes related to antioxidant and xenobiotic-metabolizing activities. This compensatory antioxidant response against increased lipid peroxidation could be mediated via activation of Nrf2 [26]. A previous study [58] has shown that dietary oxidized lipids induce the expression of the Nrf2 and subsequent increase in antioxidant enzymes in intestinal mucosa. In particular, 4-HHE increases GPx2 expression in jejunum of male C57BL/6 mice [59] involved in detoxification of lipid hydroperoxides. Interestingly, we found no differences in erythrocyte GPx activity between the rats supplemented with the fish oil containing 80% DHA and those supplemented with the coconut oil (Table 5). A previous study [60] has shown that a supplementation with virgin coconut oil increases antioxidant enzymes and decreases lipid peroxidation compared to copra oil, olive oil and sunflower oil, probably related to its high amounts of polyphenols and tocopherols.

## 3. Materials and Methods

### 3.1. Ethics Statement

All procedures on animals were adhered to the European Union (EU) guidelines for the care and handling of laboratory animals (Directive 2010/63/EU). The ethical approval was obtained from the Spanish National Research Council (CSIC) Subcommittee on Bioethical Issues and the regional Catalan authorities (reference number DAAM7921).

### 3.2. Animals and Experimental Design

A total of forty 22 weeks-old male Sprague–Dawley rats weighing about 400 g were purchased (Hsd:SD, Envigo, Indianapolis, IN, USA). The rats were housed in pairs in Makrolon cages (425 × 265 × 180 mm) under controlled conditions of temperature (22 ± 2 °C), humidity (60%), and a 12-h artificial light/dark cycle.

After acclimatization, the rats were divided into four groups (10 per group) and fed a standard diet (Table S3; Teklad Global 14% Protein Rodent Maintenance Diet, Envigo, Indianapolis, IN, USA), supplemented with coconut oil, soybean oil, EPA/DHA 1:1 or fish oil containing 80% DHA for 10 weeks. The rats were given free access to food and water throughout the study.

The oils were administered twice a week by oral gavage using a gastric probe at a dose of 0.8 mL oil/kg body weight. The coconut oil and the soybean oil were supplied by Fauser Vitaquellwerk KG (Hamburg, Germany) and Clearspring Ltd (London, UK), respectively. Fish oil containing EPA/DHA 1:1 ratio and 50% EPA + DHA of total fatty acids was obtained by combining fish oils AFAMPES 121 EPA (210 mg DHA/g fish oil; 55 mg EPA/g fish oil; AFAMSA, Vigo, Spain) and Omega-3 RX (250 mg DHA/g fish oil; 460 mg EPA/g fish oil; EnerZona, Milan, Italy). Fish oil containing 80% DHA was purchased from IFIGEN-EQUIP 98 S.L. (Barcelona, Spain). The dose of DHA supplement used in the present study was increased about 1.7-fold the safe dose suggested by the European Food Safety Authority for European general population [61]. The dose translation was performed as described in [62] based on body surface area.

### 3.3. Fatty Acid Composition and Peroxide Content of the Oils

Fatty acid composition of the soybean oil, the fish oil containing EPA/DHA 1:1 and the fish oil containing 80% DHA was measured according to the method developed by Lepage and Roy [63] using a gas chromatography coupled to a flame ionization detector technique (GC/FID; Clarus 500; PerkinElmer, Shelton, CT, USA), as previously described [18,64]. Because coconut oil is largely made up of medium-chain fatty acids (C6–12) and our GC/FID technique is not suitable for measuring this type of lipids, the fatty acid composition of coconut oil was assumed to be the one described by Lal et al. [65]. Fatty acid composition of the oils is described in the Table S2. Briefly, lauric acid (12:0) was the predominant fatty acid in the coconut oil (50% of total fatty acids), ω-6 LA in the soybean oil (50%), both EPA and DHA in the fish oil containing EPA/DHA 1:1 (27% EPA + 26% DHA), and DHA in the fish oil containing 80% DHA and 4% EPA.

In order to ensure that the oils were not oxidized prior to the nutritional intervention, amount of peroxide was measured according to the method developed by Chapman and Mackay [66] using a Beckman DU-640 UV-Vis spectrophotometer (Beckman Instruments Inc., Palo Alto, CA, USA). None of oils was oxidized before the beginning of the study—peroxide values below 10 mEq oxygen/kg oil.

### 3.4. Feed Intake and Biometric Data

Feed intake (g) and body weight (g) were monitored twice a week. At the end of the study, body weight gain, adiposity index (perigonadal white adipose tissue weight [g]/body weight [g] × 100) and hepatosomatic index (liver weight [g]/body weight [g] × 100) were calculated.

### 3.5. Measurement of Blood Glucose

Fasting blood glucose concentration was measured at week 0, 4, 8 and 10 of the study by means of the enzyme electrode method using an Ascensia ELITE XL blood glucometer (Bayer Consumer Care, Basel, Switzerland). Furthermore, OGTT were performed during week 9 of the study on fasting animals. Briefly, blood glucose was measured before the administration of a glucose solution (1 g/kg body weight) by oral gavage, and 15, 30, 45, 60, 90 and 120 min after glucose intake.

### 3.6. Sample Processing

After 10 weeks, the rats were fasted overnight, anesthetized intraperitoneally with ketamine and xylazine (80 and 10 mg/kg body weight, respectively) and sacrificed by exsanguination. Blood samples were taken by cardiac puncture. Subsequently, plasma was obtained by centrifugation at 850× *g* for 15 min at 4 °C. After the removal of plasma, erythrocytes were obtained by washing twice with 154 mM sodium chloride solution and centrifugation at 1300× *g* for 5 min at 4 °C. Plasma and erythrocytes samples were aliquoted, and a portion of erythrocytes was mixed with 5 mM protease inhibitor phenylmethanesulfonyl fluoride for fatty acid analysis. All samples were stored at −80 °C until use, except one aliquot of erythrocytes that was washed five times with 5 mM sodium phosphate dibasic solution and centrifuged at 15,000× *g* for 15 min at 4 °C for obtainment of erythrocyte membranes to measure membrane fluidity.

Perigonadal white adipose tissue as a marker of visceral adiposity, the liver and gastrocnemius muscle were collected, washed with 154 mM sodium chloride solution, weighted, and cut. After that, samples of perigonadal adipose tissue, the liver and gastrocnemius muscle were snap-frozen in liquid nitrogen and stored at −80 °C until use, except one part of the liver that was fixed for 24 h in 4% formaldehyde solution for histological study. Perigonadal adipose tissue samples were homogenized on ice in 200 mM sodium phosphate buffer (pH 6.25), sonicated for 1 min, and centrifuged at 1000× *g* for 10 min at 4 °C. Then, soluble fraction was carefully collected, and centrifuged at 129,000× *g* for 1 h at 4 °C. Frozen liver samples were divided into three parts. One part of the liver sample was homogenized on ice in 154 mM sodium chloride solution containing 0.1% Triton X-100 and centrifuged at 3000× *g* for 5 min at room temperature for measurements of TAG and TC. The second part was homogenized on ice in 200 mM sodium phosphate buffer (pH 6.25) and centrifuged at 129,000× *g* for 1 h at 4 °C for measurement of several oxidative stress biomarkers. The other part was homogenized on ice in 50 mM potassium phosphate buffer (pH 7.8) supplemented with 0.5 mM dithiothreitol and 1 mM EDTA, and centrifuged at 129,000× *g* for 1 h at 4 °C for measurements of GST and XO. All tissue samples were aliquoted and stored at −80 °C until use.

### 3.7. Measurements of Total Fat and Fatty Acids in Blood and Tissues

Total fat content was extracted from erythrocytes, plasma, the liver and gastrocnemius muscle, and gravimetrically quantified as described in Dasilva et al. [11]. Fatty acid composition was measured in erythrocytes, perigonadal adipose tissue and the liver as previously described [11] by means of a GC/FID technique (Clarus 500; PerkinElmer, Shelton, CT, USA). Furthermore, activities of fatty acid desaturase were calculated as the ratio between product and substrate—i.e., SCD-16 = [16:1 ω-7/16:0], SCD-18 = [18:1 ω-9/18:0], Δ5D = [20:4 ω-6/20:3 ω-6], Δ6D = [20:3 ω-6/18:2 ω-6] and Δ5/6D = [20:5 ω-3/18:3 ω-3].

### 3.8. Measurement of Erythrocyte Membrane Fluidity

Erythrocyte membrane fluidity was measured as previously described [67]. Briefly, freshly isolated erythrocyte membranes were incubated with 1 μM 1,6-diphenyl-1,3,5-hexatriene (DPH) for 1 h at room temperature under constant agitation. DPH incorporates in the hydrophobic region of the lipid bilayer, between fatty acyls. After that, the steady-state anisotropy (r) of the sample was measured using an LS55 fluorescence spectrophotometer (Perkin Elmer, Shelton, CT, USA). The membrane fluidity was calculated as the inverse value of anisotropy (1/r).

### 3.9. Measurements of Glycated Hemoglobin and Total hemoglobin

Blood glycated hemoglobin (HbA1c) was measured by means of spectrophotometry using the corresponding commercial kit (Spinreact, Girona, Spain) in a COBAS MIRA autoanalyzer (Roche Diagnostics System, Madrid, Spain). Blood hemoglobin (Hb) concentration was measured according to the Drabkin’s method [68] using a Lambda 25 UV-Vis spectrophotometer (Perkin Elmer, Shelton, CT, USA).

### 3.10. Measurements of Lipid Profile, Transaminases and Inflammatory Biomarkers

Plasma TAG, TC, HDL and LDL concentrations were measured by means of colorimetric enzymatic methods using the corresponding commercial kits (Spinreact, Girona, Spain) in a COBAS MIRA autoanalyzer (Roche Diagnostics System, Madrid, Spain). LDL/HDL ratio was also calculated. Liver TAG and TC contents were measured in homogenates, as described above.

Plasma AST and ALT activities were measured by means of spectrophotometry using the corresponding commercial kits (Spinreact, Girona, Spain) in a COBAS MIRA autoanalyzer (Roche Diagnostics System, Madrid, Spain). AST/ALT ratio was also calculated as a biomarker of the liver function [69].

Plasma TNFα and CRP concentrations were measured using the corresponding ELISA kits (Invitrogen, Waltham, MA, USA) in a PowerWave XS2 microplate spectrophotometer (Biotek Instruments Inc., Winooski, VT, USA).

### 3.11. Histological Analysis of the Liver

Fixed-liver samples were subjected to alcohol dehydration and paraffin infiltration-immersion at 52 °C. Subsequently, training paraffin block and successive 2-μm thickness sections were performed (Microm HM 355S; Thermo Fisher Scientific, Waltham, MA, USA). Sections were deposited above slides and subjected to automated hematoxylin-eosin staining (Shandon Varistain Gemini; Thermo Fisher Scientific, Waltham, MA, USA). Histological examination was done using a Leica DM750 microscope (Leica Microsystems, Wetzlar, Germany) [70]. A single observer was blinded to avoid bias.

### 3.12. Measurements of Oxidative Stress Biomarkers

#### 3.12.1. Plasma Antioxidant Capacity

Plasma non-enzymatic antioxidant capacity was assessed using the ORAC [71] and the ferric reducing ability of plasma (FRAP) assays [72]. The ORAC was measured using a Fluoroskan Ascent microplate fluorimeter (Labsystems, Helsinki, Finland). The FRAP was measured using a PowerWave XS2 microplate spectrophotometer (Biotek Instruments Inc., Winooski, VT, USA).

#### 3.12.2. Antioxidant Enzymes, Glutathione and Xanthine Oxidase

Major antioxidant enzyme systems include SOD, CAT, GPx, GR and GST, which were assessed in erythrocytes, perigonadal adipose tissue and liver. Perigonadal adipose tissue and liver play important roles in lipid metabolism and then can be especially vulnerable to oxidative stress under high exposure to unsaturated fat. GST activity was only measured in liver because it is highly expressed in this tissue for detoxification purposes of endogenous compounds (e.g., end products of lipid peroxidation) and xenobiotics. SOD and CAT activities were measured according to the methods developed by Mirsa and Fridovich [73] and Cohen et al. [74], respectively, using a Lambda 25 UV-Vis spectrophotometer (Perkin Elmer, Shelton, CT, USA). One unit of SOD is defined as the amount of SOD that produces a 50% inhibition of the transformation of epinephrine to adrenochrome per min at pH 10.2 at 25 °C. One unit of CAT is defined as the amount of CAT that decomposes 1 µmol of hydrogen peroxide per min at pH 7.5 at 25 °C. GPx and GR activities were measured according to the method developed by Wheeler et al. [75] using a COBAS MIRA autoanalyzer (Roche Diagnostics System, Madrid, Spain). One unit of GPx is defined as the amount of GPx that oxidizes 1 µmol of NADPH per min at pH 7 at 25 °C. One unit of GR is defined as the amount of GR that produces 1 µmol of NADP+ per min at pH 7.4 at 25 °C. GST activity was measured according to the method developed by Habig et al. [76] using a COBAS MIRA autoanalyzer (Roche Diagnostics System, Madrid, Spain). One unit of GST is defined as the amount of GST that conjugates 1 µmol of 1-chloro-2,4-dinitrobenze with GSH per min at pH 6.25 at 25 °C.

GSH and GSSG in plasma, erythrocytes, perigonadal adipose tissue and the liver were measured according to the method developed by Hissin and Hilf [77] using an LS55 fluorescence spectrophotometer (Perkin Elmer, Shelton, CT, USA). GSSG/GSH ratio was also calculated as a biomarker of the redox state.

XO activity in the liver was measured according to the method described by Maia and Mira [78] using a Lambda 25 UV-Vis spectrophotometer (Perkin Elmer, Shelton, CT, USA). One unit of XO is defined as the amount of XO that produces 1 µmol of uric acid per min at pH 7.8 at 25 °C.

#### 3.12.3. Lipid Peroxidation and Protein Carbonylation

Lipid peroxidation was assessed in circulation and tissues. Plasma oxidized LDL concentration was measured using the corresponding ELISA kit (MyBioSource Inc., San Diego, CA, USA) in a PowerWave XS2 microplate spectrophotometer (Biotek Instruments Inc., Winooski, VT, USA). Conjugated diene hydroperoxide amount in perigonadal adipose tissue and the liver was measured as previously described [79] using a Beckman DU-640 UV-Vis spectrophotometer (Beckman Instruments Inc., Palo Alto, CA, USA). TBARS amount in plasma, erythrocytes, perigonadal adipose tissue and the liver was measured according to the method developed by Buege and Aust [80] with modifications described by Richard et al. [81] using an LS55 fluorescence spectrophotometer (Perkin Elmer, Shelton, CT, USA).

Protein carbonylation in plasma and the liver was measured as previously described [79] by labeling carbonyl-modified proteins with fluorescein-5-thiosemicarbazide (FTSC) and resolving and quantified them using a one-dimensional sodium dodecyl sulfate-polyacrylamide gel electrophoresis.

### 3.13. Statistical Analysis

The statistical analysis was performed using SPSS v.26 software (IBM, Chicago, IL, USA). The results are expressed as mean and standard deviation, except the histological data that is expressed in frequencies (%). The Shapiro–Wilk test was used to test for normality of data. Groups were then compared by means of the one-way analysis of variance followed by the Scheffé post hoc test or the non-parametric Kruskal–Wallis test followed by the Mann–Whitney U test. Frequencies were compared by means of contingency tables using *χ*^2^ statistics. The level of statistical significance was set at *p*-value < 0.05.

## 4. Conclusions

**Figure 2:**
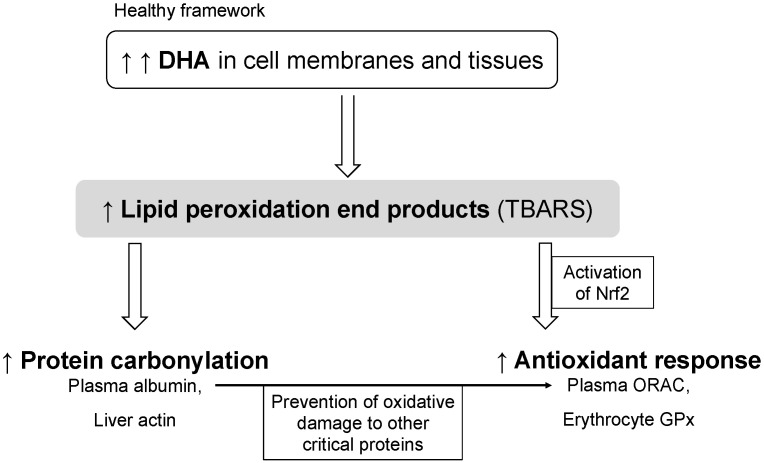
A proposed model of antioxidant response induced by high doses of DHA. Abbreviations: DHA, docosahexaenoic acid; TBARS, thiobarbituric acid-reactive substances; Nrf2, nuclear factor-erythroid 2-related factor 2; ORAC, oxygen radical absorbance capacity; GPx, glutathione peroxidase.

The supplementation with fish oil rich in DHA beneficially modulated total fat and cholesterol in healthy rats compared to the coconut oil and the soybean oil. The high dose of DHA increased the amount of DHA and EPA in erythrocytes and tissues, with a concomitant decrease in ARA. The accumulation of DHA promoted lipid peroxidation and protein carbonylation. In our healthy framework, lipid peroxidation and protein carbonylation of plasma albumin and probably actin in liver after receiving fish oil rich in DHA also enhances specific aspects of the antioxidant response, as seen from increased ORAC and erythrocyte GPx, against end products of lipid peroxidation to prevent oxidative damage to other critical proteins, such as LDL particles in plasma (Figure 2).

## Figures and Tables

**Table 1:** Glucose metabolism, plasma lipid profile, ectopic fat, transaminases, erythrocyte membrane properties and inflammatory biomarkers at the end of the study.

	Coconut	Soybean	EPA/DHA 1:1	80% DHA	*p*-Value
Glucose metabolism					
Blood glucose (mmol/L)	5.1 ± 0.3	5.5 ± 0.4 ^a^	5.0 ± 0.2 ^b^	5.0 ± 0.2 ^b^	0.008 ^†^
Blood HbA1c (%)	2.9 ± 0.1	3.0 ± 0.3	3.2 ± 0.2	3.2 ± 0.3	0.035 *
Plasma lipid profile					
Fat content (%)	0.52 ± 0.04	0.45 ± 0.03	0.44 ± 0.03 ^a^	0.37 ± 0.04 ^a,b^	0.033 *
TAG (mmol/L)	0.7 ± 0.1	0.7 ± 0.2	0.7 ± 0.2	0.7 ± 0.2	NS *
TC (mmol/L)	2.7 ± 0.3	2.5 ± 0.4	2.4 ± 0.2	2.1 ± 0.2 ^a,b^	0.001 *
HDL (mmol/L)	1.80 ± 0.21	1.69 ± 0.21	1.62 ± 0.12 ^a^	1.47 ± 0.15 ^a,b,c^	0.004 ^†^
LDL (mmol/L)	0.45 ± 0.08	0.35 ± 0.13 ^a^	0.45 ± 0.05 ^b^	0.39 ± 0.09	0.027 ^†^
LDL/HDL ratio	0.25 ± 0.03	0.20 ± 0.06 ^a^	0.28 ± 0.03 ^a,b^	0.27 ± 0.06 ^b^	0.003 ^†^
Ectopic fat					
Liver fat content (%)	5.36 ± 0.19	5.13 ± 0.35	4.92 ± 0.31 ^a^	5.12 ± 0.32 ^c^	0.019 *
Liver TAG (µmol/g tissue)	11.2 ± 1.1	10.9 ± 1.7	11.0 ± 1.3	10.9 ± 1.3	NS ^†^
Liver TC (µmol/g tissue)	4.7 ± 0.9	4.8 ± 1.1	5.8 ± 0.6	5.6 ± 0.6	0.014 *
Muscle fat content (%)	2.13 ± 0.67	2.27 ± 0.53	2.17 ± 0.43	1.64 ± 0.17 ^a,b^	0.014 *
Transaminases					
Plasma AST (U/L)	91.2 ± 29.4	85.8 ± 31.7	110.2 ± 15.5	120.7 ± 21.0	0.023 *
Plasma ALT (U/L)	43.9 ± 15.9	43.8 ± 11.3	49.0 ± 12.2	53.8 ± 23.8	NS ^†^
Plasma AST/ALT ratio	2.13 ± 0.55	1.92 ± 0.34	2.39 ± 0.71	2.49 ± 0.88	NS ^†^
Erythrocyte membrane properties					
Fat content (%)	2.05 ± 0.19	2.04 ± 0.20	1.98 ± 0.26	1.98 ± 0.23	NS *
Fluidity (AU)	4.6 ± 0.9	5.1 ± 0.9	4.9 ± 0.9	4.9 ± 0.8	NS ^†^
Inflammatory biomarkers					
Plasma TNFα (pg/mL)	21.6 ± 5.7	18.3 ± 8.8	27.9 ± 6.8 ^a,b^	30.6 ± 10.2 ^a,b^	0.004 ^†^
Plasma CRP (μg/mL)	390 ± 30	431 ± 56	380 ± 40	388 ± 47	NS *

Values are expressed as mean ± standard deviation, *n* = 10 rats/group. Abbreviations: EPA, eicosapentaenoic acid; DHA, docosahexaenoic acid; HbA1c, glycated hemoglobin; NS, not significant; TAG, triacylglycerol; TC, total cholesterol; HDL, high-density lipoprotein cholesterol; LDL, low-density lipoprotein cholesterol; AST, aspartate aminotransferase; ALT, alanine aminotransferase; AU, arbitrary unit; TNFα, tumor necrosis factor α; CRP, C-reactive protein. * *p*-value was calculated by the one-way analysis of variance followed by the Scheffé post hoc test. ^†^ *p*-value was calculated by the non-parametric Kruskal–Wallis test followed by the Mann–Whitney U test. The level of statistical significance was set at *p*-value < 0.05. ^a^ vs. coconut, ^b^ vs. soybean, ^c^ vs. EPA/DHA 1:1.

**Table 2:** Fatty acids in erythrocytes at the end of the study.

FA	Coconut	Soybean	EPA/DHA 1:1	80% DHA	*p*-Value
14:0	0.31 ± 0.08	0.26 ± 0.05	0.34 ± 0.22	0.20 ± 0.02	NS
15:0	0.37 ± 0.03	0.32 ± 0.06	0.37 ± 0.04	0.34 ± 0.03	0.021
16:0	30.02 ± 0.70	29.59 ± 0.86	30.89 ± 1.02 ^b^	30.79 ± 0.78 ^b^	0.003
16:1 ω-7	0.51 ± 0.08	0.52 ± 0.11	0.53 ± 0.16	0.42 ± 0.06	NS
17:0	0.47 ± 0.05	0.46 ± 0.07	0.51 ± 0.04	0.39 ± 0.14 ^c^	0.033
18:0	11.85 ± 0.34	12.08 ± 0.37	11.58 ± 0.28 ^b^	12.11 ± 0.33 ^c^	0.003
18:1 ω-9	5.68 ± 0.30	5.79 ± 0.19	5.44 ± 0.77	5.60 ± 0.24	NS
18:1 ω-7	3.47 ± 0.16	3.30 ± 0.14	3.67 ± 1.02	2.95 ± 0.14 ^c^	0.034
18:2 ω-6	8.91 ± 0.46	9.24 ± 0.19	10.22 ± 0.4 ^a,b^	10.94 ± 0.43 ^a,b,c^	<0.001
18:3 ω-6	0.05 ± 0.04	0.06 ± 0.04	0.01 ± 0.03 ^b^	ND ^a,b^	<0.001
18:3 ω-3	0.04 ± 0.05	0.07 ± 0.04	0.07 ± 0.04	ND ^b,c^	<0.001
20:1 ω-9	0.17 ± 0.03	0.18 ± 0.04	0.18 ± 0.07	0.15 ± 0.02	NS
20:2 ω-6	0.33 ± 0.04	0.34 ± 0.04	0.33 ± 0.05	0.33 ± 0.04	NS
20:3 ω-6	0.41 ± 0.05	0.39 ± 0.06	0.47 ± 0.05 ^b^	0.54 ± 0.06 ^a,b^	0.015
20:4 ω-6	22.71 ± 0.45	22.47 ± 0.74	19.69 ± 0.87 ^a,b^	18.55 ± 0.85 ^a,b,c^	<0.001
22:1 ω-9	0.33 ± 0.10	0.34 ± 0.09	0.26 ± 0.06	0.33 ± 0.09	NS
20:5 ω-3	0.21 ± 0.04	0.22 ± 0.03	0.86 ± 0.33 ^a,b^	0.87 ± 0.21 ^a,b^	<0.001
24:0	1.06 ± 0.11	1.23 ± 0.14 ^a^	1.16 ± 0.05	1.19 ± 0.12	0.013
22:4 ω-6	2.68 ± 0.22	2.67 ± 0.15	1.40 ± 0.18 ^a,b^	1.07 ± 0.15 ^a,b,c^	<0.001
24:1 ω-9	0.55 ± 0.04	0.68 ± 0.14 ^a^	0.60 ± 0.07	0.55 ± 0.06 ^b^	0.006
22:5 ω-6	1.09 ± 0.11	1.04 ± 0.08	0.61 ± 0.05 ^a,b^	0.63 ± 0.06 ^a,b^	<0.001
22:5 ω-3	1.54 ± 0.08	1.69 ± 0.17	2.56 ± 0.14 ^a,b^	1.86 ± 0.12 ^a,c^	<0.001
22:6 ω-3	1.83 ± 0.19	1.86 ± 0.1	3.30 ± 0.2 ^a,b^	5.21 ± 0.56 ^a,b,c^	<0.001
SAT	48.12 ± 0.85	47.87 ± 0.83	48.59 ± 1.09	48.87 ± 0.99	NS
MUFA	11.51 ± 0.37	11.4 ± 0.34	11.29 ± 0.42	10.6 ± 0.3 ^a,b,c^	<0.001
PUFA	40.36 ± 0.9	40.72 ± 0.87	40.13 ± 1.35	40.53 ± 1.09	NS
ω-3	3.63 ± 0.22	3.85 ± 0.24	6.79 ± 0.51 ^a,b^	7.93 ± 0.8 ^a,b,c^	<0.001
ω-6	36.18 ± 0.81	36.2 ± 0.73	32.73 ± 1.08 ^a,b^	32.05 ± 0.97 ^a,b^	<0.001
ω-6/ω-3 ratio	10.01 ± 0.59	9.44 ± 0.58	4.84 ± 0.34 ^a,b^	4.08 ± 0.48 ^a,b,c^	<0.001
SCD-16 = [16:1 ω-7/16:0]	0.02 ± 0.00	0.02 ± 0.00	0.02 ± 0.00	0.01 ± 0.00	0.017
SCD-18 = [18:1 ω-9/18:0]	0.48 ± 0.02	0.48 ± 0.02	0.47 ± 0.07	0.46 ± 0.02	NS
Δ5D = [20:4 ω-6/20:3 ω-6]	56.14 ± 6.69	59.32 ± 9.01	42.35 ± 3.58 ^a,b^	35.16 ± 5.36 ^a,b^	<0.001
Δ6D = [20:3 ω-6/18:2 ω-6]	0.05 ± 0.01	0.04 ± 0.01	0.05 ± 0.00	0.05 ± 0.00	NS
Δ5/6D = [20:5 ω-3/18:3 ω-3]	2.40 ± 0.78	2.62 ± 0.66	11.01 ± 4.65	ND	

Values are expressed as mean ± standard deviation (mg/100 mg of total FA), *n* = 10 rats/group. Abbreviations: FA, fatty acid; NS, not significant; ND, non-detected; SAT, saturated fatty acids; MUFA, monounsaturated fatty acids; PUFA, polyunsaturated fatty acids. *p*-value was calculated by the one-way analysis of variance followed by the Scheffé post hoc test. The level of statistical significance was set at *p*-value < 0.05. ^a^ vs. coconut, ^b^ vs. soybean, ^c^ vs. EPA/DHA 1:1.

**Table 3:** Fatty acids in perigonadal adipose tissue at the end of the study.

FA	Coconut	Soybean	EPA/DHA 1:1	80% DHA	*p*-Value
14:0	1.15 ± 0.17	0.94 ± 0.11 ^a^	0.98 ± 0.11 ^a^	0.87 ± 0.09 ^a^	<0.001
15:0	0.33 ± 0.04	0.30 ± 0.03	0.33 ± 0.03	0.32 ± 0.04	NS
16:0	19.22 ± 1.78	19.25 ± 1.79	19.41 ± 1.75	18.52 ± 1.62	NS
16:1 ω-9	0.59 ± 0.05	0.59 ± 0.04	0.57 ± 0.03	0.62 ± 0.06	NS
16:1 ω-7	3.86 ± 1.21	3.41 ± 0.73	3.95 ± 1.52	3.28 ± 1.17	NS
17:0	0.24 ± 0.03	0.24 ± 0.02	0.25 ± 0.03	0.25 ± 0.03	NS
18:0	2.38 ± 0.30	2.45 ± 0.24	2.43 ± 0.38	2.47 ± 0.32	NS
18:1 ω-9	23.21 ± 0.40	23.44 ± 0.4	23.01 ± 0.46	22.88 ± 0.44	0.033
18:1 ω-7	3.88 ± 0.32	3.77 ± 0.22	3.87 ± 0.19	3.6 ± 0.22	NS
18:2 ω-6	40.37 ± 2.02	41.15 ± 1.71	39.99 ± 1.5	41.38 ± 1.44	NS
18:3 ω-6	0.11 ± 0.01	0.10 ± 0.02	0.10 ± 0.01	0.09 ± 0.01 ^a^	0.022
20:0	0.07 ± 0.02	0.08 ± 0.02	0.10 ± 0.03	0.08 ± 0.01	NS
18:3 ω-3	1.99 ± 0.32	2.01 ± 0.27	1.93 ± 0.30	1.98 ± 0.18	NS
20:1 ω-9	0.31 ± 0.08	0.29 ± 0.04	0.32 ± 0.07	0.29 ± 0.05	NS
20:1 ω-7	0.37 ± 0.11	0.36 ± 0.07	0.42 ± 0.12	0.36 ± 0.08	NS
20:2 ω-6	0.31 ± 0.09	0.28 ± 0.08	0.29 ± 0.09	0.32 ± 0.1	NS
20:3 ω-6	0.18 ± 0.05	0.14 ± 0.05	0.16 ± 0.05	0.19 ± 0.07	NS
20:4 ω-6	0.80 ± 0.21	0.69 ± 0.20	0.69 ± 0.29	0.67 ± 0.24	NS
20:5 ω-3	0.02 ± 0.03	ND	0.2 ± 0.04 ^a,b^	0.12 ± 0.03 ^a,b,c^	<0.001
22:4 ω-6	0.24 ± 0.06	0.20 ± 0.05	0.18 ± 0.07	0.19 ± 0.07	NS
22:5 ω-3	0.20 ± 0.08	0.16 ± 0.06	0.33 ± 0.08 ^a,b^	0.34 ± 0.09 ^a,b^	<0.001
22:6 ω-3	0.19 ± 0.07	0.17 ± 0.06	0.5 ± 0.12 ^a,b^	1.18 ± 0.25 ^a,b,c^	<0.001
SAT	23.38 ± 2.09	23.26 ± 2.11	23.49 ± 2.18	22.5 ± 2	NS
MUFA	32.22 ± 1.18	31.85 ± 0.73	32.13 ± 1.42	31.04 ± 1.41	NS
PUFA	44.40 ± 2.44	44.89 ± 2.30	44.37 ± 1.88	46.46 ± 1.65	NS
ω-3	2.39 ± 0.48	2.33 ± 0.37	2.96 ± 0.50 ^b^	3.63 ± 0.43 ^a,b,c^	0.003
ω-6	42 ± 2.23	42.56 ± 1.98	41.42 ± 1.58	42.83 ± 1.58	NS
ω-6/ω-3 ratio	19.47 ± 3.43	19.94 ± 2.30	15.61 ± 2.28 ^a,b^	13.20 ± 1.66 ^a,b^	0.006
SCD-16 = [16:1 ω-7/16:0]	0.20 ± 0.07	0.18 ± 0.05	0.21 ± 0.10	0.18 ± 0.07	NS
SCD-18 = [18:1 ω-9/18:0]	9.98 ± 1.24	9.73 ± 0.87	9.80 ± 1.84	9.51 ± 1.46	NS
Δ5D = [20:4 ω-6/20:3 ω-6]	4.66 ± 0.79	4.99 ± 0.57	4.28 ± 0.51	3.73 ± 0.60 ^a,b^	<0.001
Δ6D = [20:3 ω-6/18:2 ω-6]	0.004 ± 0.001	0.003 ± 0.001	0.004 ± 0.001	0.004 ± 0.001	NS
Δ5/6D = [20:5 ω-3/18:3 ω-3]	0.008 ± 0.013	0.000 ± 0.000	0.103 ± 0.026 ^a,b^	0.062 ± 0.014 ^a,b,c^	<0.001

Values are expressed as mean ± standard deviation (mg/100 mg of total FA), *n* = 10 rats/group. Abbreviations: FA, fatty acid; NS, not significant; ND, non-detected; SAT, saturated fatty acids; MUFA, monounsaturated fatty acids; PUFA, polyunsaturated fatty acids. *p*-value was calculated by the one-way analysis of variance followed by the Scheffé post hoc test. The level of statistical significance was set at *p*-value < 0.05. ^a^ vs. coconut, ^b^ vs. soybean, ^c^ vs. EPA/DHA 1:1.

**Table 4:** Fatty acids in the liver at the end of the study.

FA	Coconut	Soybean	EPA/DHA 1:1	80% DHA	*p*-Value
14:0	0.34 ± 0.12	0.24 ± 0.03	0.24 ± 0.06	0.23 ± 0.07	NS
15:0	0.22 ± 0.06	0.20 ± 0.02	0.19 ± 0.02	0.20 ± 0.02	NS
16:0	22.29 ± 1.10	21.58 ± 0.78	22.66 ± 0.58	23.07 ± 1.18 ^b^	0.009
16:1 ω-7	1.19 ± 0.34	0.92 ± 0.16	0.87 ± 0.16	0.86 ± 0.29 ^a^	0.042
17:0	0.39 ± 0.05	0.40 ± 0.06	0.38 ± 0.03	0.37 ± 0.04	NS
18:0	13.08 ± 1.28	14.26 ± 1.19	13.79 ± 0.81	13.72 ± 1.54	NS
18:1 ω-9	5.74 ± 2.26	5.61 ± 0.56	5.07 ± 0.48	4.81 ± 1.31	NS
18:1 ω-7	3.82 ± 0.45	3.52 ± 0.25	3.19 ± 0.23 ^a^	2.91 ± 0.38 ^a,b^	<0.001
18:2 ω-6	20.59 ± 1.17	20.16 ± 1.29	20.94 ± 1.02	22.37 ± 1.15 ^b^	0.014
18:3 ω-6	0.24 ± 0.05	0.25 ± 0.03	0.15 ± 0.03 ^a,b^	0.12 ± 0.02 ^a,b^	<0.001
20:0	0.04 ± 0.04	0.08 ± 0.01	0.00 ± 0.00 ^a,b^	0.00 ± 0.00 ^a,b^	<0.001
18:3 ω-3	0.38 ± 0.06	0.35 ± 0.05	0.31 ± 0.06	0.33 ± 0.11	NS
20:1 ω-9	0.12 ± 0.03	0.12 ± 0.02	0.10 ± 0.01	0.10 ± 0.04	NS
20:1 ω-7	0.25 ± 0.04	0.26 ± 0.04	0.26 ± 0.07	0.23 ± 0.04	NS
20:2 ω-6	0.35 ± 0.07	0.36 ± 0.07	0.29 ± 0.04	0.30 ± 0.03	0.021
20:3 ω-6	0.42 ± 0.09	0.41 ± 0.12	0.54 ± 0.08 ^b^	0.66 ± 0.09 ^a,b^	<0.001
20:4 ω-6	22.01 ± 1.60	22.71 ± 0.94	19.26 ± 1.32 ^a,b^	16.15 ± 2.31 ^a,b^	<0.001
22:1 ω-9	0.07 ± 0.04	0.1 ± 0.02	0.00 ± 0.00 ^a,b^	0.12 ± 0.02 ^c^	0.025
23:0	0.14 ± 0.02	0.14 ± 0.02	0.13 ± 0.03	0.13 ± 0.02	NS
20:5 ω-3	0.23 ± 0.08	0.21 ± 0.03	0.91 ± 0.2 ^a,b^	1.09 ± 0.23 ^a,b^	<0.001
24:0	0.43 ± 0.04	0.42 ± 0.06	0.46 ± 0.03	0.45 ± 0.06	NS
22:4 ω-6	0.61 ± 0.06	0.58 ± 0.03	0.30 ± 0.02^a,b^	0.21 ± 0.04 ^a,b,c^	<0.001
24:1 ω-9	0.19 ± 0.17	0.13 ± 0.02	0.15 ± 0.01	0.15 ± 0.02	NS
22:5 ω-6	0.48 ± 0.15	0.41 ± 0.08	0.17 ± 0.02 ^a,b^	0.21 ± 0.06 ^a,b^	0.020
22:5 ω-3	1.23 ± 0.14	1.25 ± 0.14	1.53 ± 0.19 ^a,b^	1.02 ± 0.13 ^b,c^	<0.001
22:6 ω-3	5.14 ± 0.70	5.35 ± 0.29	8.10 ± 0.51 ^a,b^	10.19 ± 1.35 ^a,b,c^	<0.001
SAT	36.93 ± 1.25	37.33 ± 1.06	37.84 ± 0.63	38.17 ± 1.04 ^a^	0.032
MUFA	11.40 ± 2.60	10.65 ± 0.83	9.65 ± 0.75	9.18 ± 1.46 ^a,b^	0.012
PUFA	51.67 ± 1.40	52.02 ± 1.08	52.51 ± 0.58	52.64 ± 0.80	NS
ω-3	6.97 ± 0.69	7.16 ± 0.32	10.86 ± 0.72 ^a,b^	12.63 ± 1.57 ^a,b,c^	<0.001
ω-6	44.69 ± 0.88	44.87 ± 1.11	41.65 ± 0.87 ^a,b^	40.01 ± 1.78 ^a,b,c^	<0.001
ω-6/ω-3 ratio	6.42 ± 0.53	6.06 ± 0.29	3.79 ± 0.29 ^a,b^	3.07 ± 0.46 ^a,b,c^	<0.001
SCD-16 = [16:1 ω-7/16:0]	0.05 ± 0.01	0.04 ± 0.01	0.04 ± 0.01	0.03 ± 0.01 ^a^	0.012
SCD-18 = [18:1 ω-9/18:0]	0.50 ± 0.18	0.40 ± 0.07	0.37 ± 0.06	0.39 ± 0.13	NS
Δ5D = [20:4 ω-6/20:3 ω-6]	57.39 ± 13.78	59.20 ± 13.66	35.12 ± 6.98 ^a,b^	25.50 ± 5.64 ^a,b^	0.035
Δ6D = [20:3 ω-6/18:2 ω-6]	0.02 ± 0.01	0.02 ± 0.01	0.03 ± 0.00	0.03 ± 0.00	NS
Δ5/6D = [20:5 ω-3/18:3 ω-3]	0.54 ± 0.17	0.63 ± 0.12	2.98 ± 0.81 ^a,b^	3.63 ± 0.83 ^a,b^	<0.001

Values are expressed as mean ± standard deviation (mg/100 mg of total FA), *n* = 10 rats/group. Abbreviations: FA, fatty acid; NS, not significant; ND, non-detected; SAT, saturated fatty acids; MUFA, monounsaturated fatty acids; PUFA, polyunsaturated fatty acids. *p*-value was calculated by the one-way analysis of variance followed by the Scheffé post hoc test. The level of statistical significance was set at *p*-value < 0.05. ^a^ vs. coconut, ^b^ vs. soybean, ^c^ vs. EPA/DHA 1:1.

**Table 5:** Biomarkers of oxidative stress in blood at the end of the study.

	Coconut	Soybean	EPA/DHA 1:1	80% DHA	*p*-Value
Plasma					
ORAC (µmol TE/mL)	14.6 ± 2.7	12.5 ± 2.8	16.3 ± 2.6 ^b^	17.0 ± 1.9 ^a,b^	0.004 ^†^
FRAP (mmol TE/L)	0.12 ± 0.02	0.12 ± 0.04	0.11 ± 0.02	0.11 ± 0.03	NS *
GSH (nmol/mL)	12.1 ± 3.0	10.8 ± 4.1	7.6 ± 3.0	9.7 ± 4.1	NS *
GSSG (nmol/mL)	37.2 ± 4.1	37.2 ± 4.3	40.5 ± 4.6	38.9 ± 3.0	NS *
GSSG/GSH ratio	3.24 ± 0.80	3.80 ± 1.08	5.25 ± 1.33 ^a,b^	4.72 ± 1.66	0.012 ^†^
Albumin carbonylation index	2.37 ± 0.29	2.14 ± 0.33	2.66 ± 0.65	2.93 ± 0.46 ^a,b^	0.002 *
Oxidized LDL (μg/mL)	0.16 ± 0.03	0.15 ± 0.03	0.16 ± 0.03	0.18 ± 0.03	NS ^†^
TBARS (nmol MDA Eq/mL)	0.77 ± 0.18	0.99 ± 0.11	0.69 ± 0.20	1.04 ± 0.40 ^c^	0.011 *
Erythrocytes					
SOD (U/g Hb)	2631 ± 727	2731 ± 971	2415 ± 489	2470 ± 389	NS *
CAT (mmol/g Hb)	47.1 ± 11.5	43.0 ± 14.2	43.9 ± 7.2	52.5 ± 6.9	NS *
GPx (U/g Hb)	132 ± 31	116 ± 36	113 ± 22	164 ± 45 ^b,c^	0.011 *
GR (U/g Hb)	0.27 ± 0.12	0.26 ± 0.09	0.24 ± 0.07	0.21 ± 0.07	NS ^†^
GSH (µmol/g Hb)	1.85 ± 0.70	1.43 ± 0.67	1.50 ± 0.41	1.74 ± 0.63	NS *
GSSG (µmol/g Hb)	1.18 ± 0.40	1.06 ± 0.47	1.11 ± 0.21	1.20 ± 0.33	NS *
GSSG/GSH ratio	0.66 ± 0.19	0.78 ± 0.24	0.82 ± 0.34	0.74 ± 0.22	NS *
TBARS (nmol MDA Eq/g Hb)	0.24 ± 0.11	0.32 ± 0.27	0.52 ± 0.28	0.73 ± 0.19 ^a,b^	<0.001 *

Values are expressed as mean ± standard deviation, *n* = 10 rats/group. Abbreviations: EPA, eicosapentaenoic acid; DHA, docosahexaenoic acid; ORAC, oxygen radical absorbance capacity; TE, Trolox equivalent; NS, not significant; FRAP, ferric reducing ability of plasma; GSH, reduced glutathione; GSSG, oxidized glutathione; oxidized LDL, oxidized low-density lipoprotein; TBARS, thiobarbituric acid-reactive substances; MDA Eq, malondialdehyde equivalent; SOD, superoxide dismutase; Hb, hemoglobin; CAT, catalase; GPx, glutathione peroxidase; GR, glutathione reductase. * *p*-value was calculated by the one-way analysis of variance followed by the Scheffé post hoc test. ^†^ *p*-value was calculated by the non-parametric Kruskal–Wallis test followed by the Mann–Whitney U test. The level of statistical significance was set at *p*-value < 0.05. ^a^ vs. coconut, ^b^ vs. soybean, ^c^ vs. EPA/DHA 1:1.

**Table 6:** Biomarkers of oxidative stress in tissues at the end of the study.

	Coconut	Soybean	EPA/DHA 1:1	80% DHA	*p*-Value
Perigonadal adipose tissue					
SOD (U/g tissue)	226 ± 45	237 ± 44	236 ± 61	225 ± 36	NS *
CAT (mmol/g tissue)	0.11 ± 0.02	0.09 ± 0.01	0.11 ± 0.02	0.10 ± 0.03	NS ^†^
GPx (U/g tissue)	0.54 ± 0.20	0.58 ± 0.18	0.50 ± 0.16	0.41 ± 0.20	NS *
GR (U/g tissue)	0.40 ± 0.07	0.46 ± 0.10	0.39 ± 0.11	0.35 ± 0.08 ^b^	0.047 ^†^
GSH (nmol/g tissue)	0.93 ± 0.23	1.05 ± 0.15	1.03 ± 0.19	0.91 ± 0.14	NS *
GSSG (nmol/g tissue)	4.43 ± 0.58	4.56 ± 0.93	4.05 ± 0.52	4.53 ± 1.05	NS *
GSSG/GSH ratio	5.10 ± 1.78	4.46 ± 1.26	4.03 ± 0.67	5.11 ± 1.44	NS ^†^
Conjugated dienes (mmol hydroperoxides/kg lipid)	9.87 ± 1.43	8.83 ± 2.03	10.33 ± 2.75	10.83 ± 2.86	NS *
TBARS (nmol MDA Eq/g tissue)	2.41 ± 2.16	3.18 ± 2.51	4.95 ± 2.47 ^a^	4.99 ± 2.64 ^a^	0.046 ^†^
Liver					
SOD (U/g tissue)	6411 ± 631	6879 ± 1046	6577 ± 872	6083 ± 829	NS *
CAT (mmol/g tissue)	10.8 ± 1.3	11.1 ± 0.5	11.4 ± 1.2	11.8 ± 0.7	NS *
GPx (U/g tissue)	29.5 ± 5.2	25.1 ± 2.9	26.8 ± 2.9	26.0 ± 3.2	NS *
GR (U/g tissue)	6.80 ± 1.19	7.62 ± 2.34	7.78 ± 2.00	7.53 ± 1.73	NS *
GST (U/g tissue)	113 ± 11	115 ± 22	104 ± 21	103 ± 16	NS *
GSH (µmol/g tissue)	2.22 ± 0.55	2.22 ± 0.59	2.09 ± 0.52	2.08 ± 0.60	NS ^†^
GSSG (µmol/g tissue)	1.47 ± 0.20	1.38 ± 0.23	1.30 ± 0.16	1.23 ± 0.11	0.048 *
GSSG/GSH ratio	0.71 ± 0.25	0.65 ± 0.20	0.66 ± 0.21	0.63 ± 0.16	NS *
XO (mU/g tissue)	96 ± 26	121 ± 36	89 ± 15	88 ± 20	0.022 *
Protein carbonylation index	2.73 ± 0.68	2.11 ± 0.57	2.43 ± 0.67	3.05 ± 0.35 ^b^	0.007 *
Conjugated dienes (mmol hydroperoxides/kg lipid)	20.7 ± 2.3	20.6 ± 1.3	21.9 ± 1.5	21.0 ± 2.8	NS *
TBARS (nmol MDA Eq/g tissue)	217 ± 72	229 ± 115	278 ± 90	320 ± 94	NS *

Values are expressed as mean ± standard deviation, *n* = 10 rats/group. Abbreviations: EPA, eicosapentaenoic acid; DHA, docosahexaenoic acid; SOD, superoxide dismutase; NS, not significant; CAT, catalase; GPx, glutathione peroxidase; GR, glutathione reductase; GSH, reduced glutathione; GSSG, oxidized glutathione; TBARS, thiobarbituric acid-reactive substances; MDA Eq, malondialdehyde equivalent; GST, glutathione S-transferase; XO, xanthine oxidase. * *p*-value was calculated by the one-way analysis of variance followed by the Scheffé post hoc test. ^†^ *p*-value was calculated by the non-parametric Kruskal–Wallis test followed by the Mann–Whitney U test. The level of statistical significance was set at *p*-value < 0.05. ^a^ vs. coconut, ^b^ vs. soybean, ^c^ vs. EPA/DHA 1:1.

## Data Availability

The data presented in this study are available on request from the corresponding author.

## Supplementary Materials

The following are available online at https://www.mdpi.com/article/10.3390/md19100555/s1, Table S1: Feed intake, biometric data and blood glucose, Figure S1: Histological analysis of the liver, Table S2: Fatty acid composition of oils, Table S3: Diet composition.
