# Supplementary material for: Effects of a Fish Oil Rich in Docosahexaenoic Acid on Cardiometabolic Risk Factors and Oxidative Stress in Healthy Rats
Source: Mar Drugs. 2021 Sep 29;19(10):555. doi: 10.3390/md19100555 (PMC8539050; doi:10.3390/md19100555)
Supplement: Supplementary file 1 [file marinedrugs-19-00555-s001.zip › marinedrugs-1402226-supplementary.pdf]

SUPPORTING INFORMATION

Effects of a Fish Oil Rich in Docosahexaenoic Acid on Cardiometabolic Risk Factors and Oxidative Stress in Healthy Rats

Bernat Miralles-Pérez [1], Lucía Méndez [2], Maria Rosa Nogués [1,*], Vanessa Sánchez-Martos [1], Àngels Fortuño-Mar [3], Sara Ramos-Romero [4,5], Mercè Hereu [4], Isabel Medina [2], and Marta Romeu [1]

[1] Functional Nutrition, Oxidation and Cardiovascular Diseases Research Group (NFOC-SALUT), Pharmacology Unit, Department of Basic Medical Sciences, Universitat Rovira i Virgili, C/ Sant Llorenç 21, E-43201 Reus, Spain

[2] Chemistry of Marine Products, Department of Food Technology, Institute of Marine Research (IIM-CSIC), C/ Eduardo Cabello 6, E-36208 Vigo, Spain

[3] Eldine Patología, C/ Plom 32, E-43006 Tarragona, Spain

[4] Department of Biological Chemistry, Institute of Advanced Chemistry of Catalonia (IQAC-CSIC), C/ Jordi Girona 18-26, E-08034 Barcelona, Spain

[5] Current address: Department of Cell Biology, Physiology & Immunology, Faculty of Biology, University of Barcelona, Avd/ Diagonal 643, E-08028 Barcelona, Spain

* Corresponding author: mariarosa.nogues@urv.cat; Tel +34-977-75-9355

**Table S1.** Feed intake, biometric data and blood glucose.					
	**Coconut**	**Soybean**	**EPA/DHA 1:1**	**80% DHA**	***p*-value**
Feed intake					
Daily feed intake (g/rat)	18.4 ± 1.7	19.2 ± 1.3	19.1 ± 0.8	19.4 ± 1.1	NS*
Biometric data					
Initial body weight (g)	403 ± 22	401 ± 18	399 ± 15	398 ±14	NS*
Final body weight (g)	435 ± 29	442 ± 21	435 ± 15	441 ± 25	NS*
Body weight gain (g)	31.8 ± 10.8	40.5 ± 12.5	35.8 ± 13.6	43.7 ± 12.3	NS*
Perigonadal adipose tissue weight (g)	5.0 ± 1.4	5.2 ± 1.1	4.7 ± 0.7	4.8 ± 1.0	NS*
Adiposity index (%)	1.2 ± 0.2	1.2 ± 0.2	1.1 ± 0.1	1.1 ± 0.2	NS*
Liver weight (g)	10.9 ± 1.4	10.8 ± 0.8	10.8 ± 0.8	11.3 ± 1.3	NS*
Hepatosomatic index (%)	2.5 ± 0.2	2.5 ± 0.1	2.5 ± 0.2	2.6 ± 0.2	NS†
Blood glucose					
Glucose at week 0 (mmol/L)	4.5 ± 0.4	4.6 ± 0.9	4.3 ± 0.6	4.7 ± 0.7	NS †
Glucose at week 4 (mmol/L)	5.1 ± 0.6	5.0 ± 0.4	4.7 ± 0.4	4.9 ± 0.4	NS*
Glucose at week 8 (mmol/L)	4.6 ± 0.6	4.6 ± 0.5	4.4 ± 0.4	4.6 ± 0.2	NS *

Values are expressed mean ± standard deviation, n = 10 rats/group. **p*-value was calculated by the one-way analysis of variance followed by Scheffé *post-hoc* test. †*p*-value was calculated by the non-parametric Kruskal–Wallis test followed by Mann–Whitney U test. The level of statistical significance was set at *p*-value < 0.05.

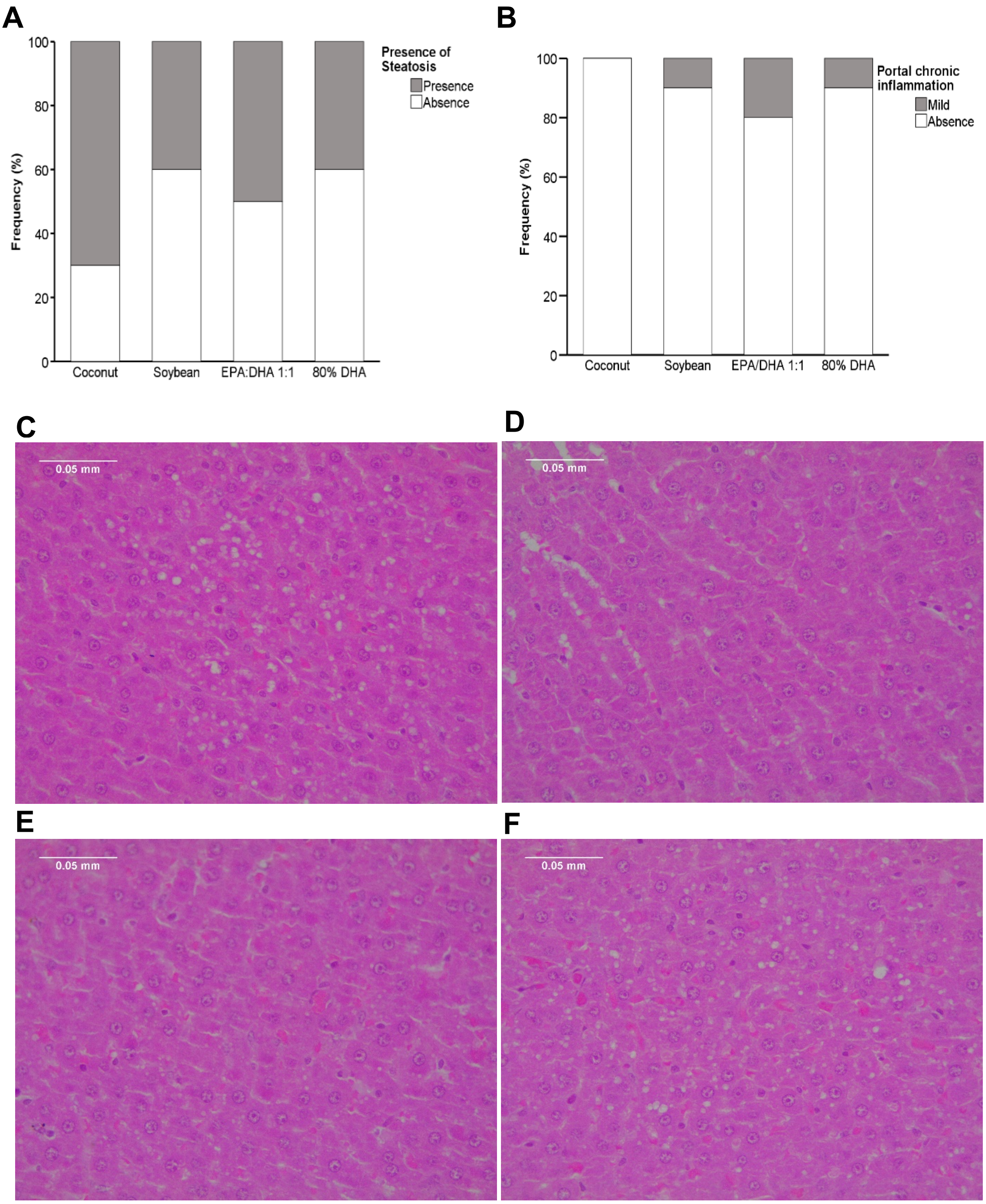

Figure S1. Histological analysis of the liver. Degree of steatosis (**A**); Portal chronic inflammation (**B**); Histological cuts of liver hematoxylin/eosin staining (400x) in rats supplemented with coconut oil (**C**) soybean oil (**D**) fish oil containing EPA/DHA 1:1 (**E**) or fish oil containing 80% DHA (**F**). Results are expressed as frequencies (%), n = 10 rats/group. No significant differences were found among groups (*p*-value > 0.05).

**Table S2**. Fatty acid composition of oils (mg/100 mg) *.				
	**Coconut**	**Soybean**	**Fish (EPA/DHA 1:1)**	**Fish (80% DHA)**
6:0	0.2–0.5			
8:0	5.4–9.5			
10:0	4.5–9.7			
12:0	44.1–51.0			
14:0	13.1–18.5	0.12 ± 0.03	1.40 ± 0.05	ND
15:0		ND	0.43 ± 0.06	ND
16:0	7.5–10.5	10.93 ± 0.13	8.39 ± 0.04	0.12 ± 0.02
16:1 ω-7		0.10 ± 0.00	1.92 ± 0.00	0.26 ± 0.01
17:0		0.16 ± 0.01	1.38 ± 0.01	0.16 ± 0.02
18:0	1.0–3.2	4.14 ± 0.08	3.74 ± 0.01	0.11 ± 0.02
18:1 ω-9	5.0–8.2	23.30 ± 0.18	8.09 ± 0.02	0.23 ± 0.03
18:1 ω-7		2.11 ± 0.01	1.88 ± 0.02	ND
18:2 ω-6	1.0–2.6	50.75 ± 0.36	1.29 ± 0.01	0.09 ± 0.02
20:0	0.2–1.5	0.33 ± 0.01	0.47 ± 0.00	ND
18:3 ω-3		7.02 ± 0.02	0.45 ± 0.03	ND
20:1 ω-9		0.20 ± 0.02	2.44 ± 0.01	ND
18:4 ω-3		ND	0.79 ± 0.01	ND
20:2 ω-6		ND	0.43 ± 0.02	ND
20:3 ω-6		ND	0.32 ± 0.01	ND
20:4 ω-6		0.49 ± 0.04	2.65 ± 0.04	0.75 ± 0.02
22:1 ω-11		ND	1.53 ± 0.00	0.10 ± 0.01
22:1 ω-9		ND	0.31 ± 0.02	ND
20:4 ω-3		ND	1.46 ± 0.03	0.26 ± 0.04
20:5 ω-3		0.10 ± 0.01	27.56 ± 0.04	3.87 ± 0.20
24:1 ω-9		ND	0.43 ± 0.01	ND
22:4 ω-6		0.25 ± 0.02	1.82 ± 0.00	1.15 ± 0.02
22:5 ω-6		ND	1.52 ± 0.10	6.18 ± 0.40
22:5 ω-3		ND	3.16 ± 0.01	4.09 ± 0.01
22:6 ω-3		ND	26.15 ± 0.03	82.63 ± 0.15

* Fatty acid composition of the soybean and the fish oils was measured according to the method described by Lepage and Roy [1], whereas fatty acid composition of coconut oil described by Lal et al. [2] was used. Abbreviations: ND, non-detected.

References

1. Lepage, G.; Roy, C.C. Direct transesterification of all classes of lipids in a one-step reaction. J. Lipid Res. 1986, 27, 114–120.

2. Lal, J.J.; Sreeranjit Kumar, C.V.; Indira, M. Coconut Palm. In Encyclopedia of Food Sciences and Nutrition; Trugo, L., Finglas, P.M., Eds.; Elsevier Science Ltd: Amsterdam, Netherlands, 2003; Vol. 1, pp. 1464–1475.

**Table S3**. Diet composition *.	
Crude protein (%)	14.3
Fat (%)	4.0
Carbohydrate (%)	48.0
Crude fiber (%)	4.1
Neutral detergent fiber (%)	18.0
Ash (%)	4.7
Energy density (kcal/g)	2.9
Calories from protein (%)	20
Calories from fat (%)	13
Calories from carbohydrate (%)	67
* The composition of diet described by the manufacturer was used (Teklad Global 14% Protein Rodent Maintenance Diet; Envigo, Indianapolis, IN, USA).	
